# A rapid and accurate method for *Helicobacter pylori* detection via integrating LAMP assay with CRISPR/Cas12b detection by one-step in one-pot

**DOI:** 10.3389/fcimb.2025.1611134

**Published:** 2025-08-26

**Authors:** Yeqian Zhang, Tao Liu, Puhua Zhang, Bo Ni, Xingang Wang, Long Bai, Wei Sun, Yujing Guan, Xiang Xia, Hui Cao, Jiayi Gu

**Affiliations:** ^1^ Department of Gastrointestinal Surgery, Renji Hospital, School of Medicine, Shanghai Jiao Tong University, Shanghai, China; ^2^ Department of Surgery, Shanghai Changhai Hospital, Shanghai, China

**Keywords:** *Helicobacter pylori*, CagA, LAMP, CRISPR/Cas12b, one-step, one-pot

## Abstract

**Introduction:**

Accurate and timely detection of *Helicobacter pylori* (HP) is crucial for the diagnosis and management of gastritis and other HP-associated gastrointestinal disorders. Conventional diagnostic methods, such as PCR and culture, require specialized equipment and expertise, limiting their applicability in resource-limited settings. There is a pressing need for a rapid, cost-effective, and user-friendly diagnostic platform for HP detection, particularly in point-of-care settings.

**Methods:**

We developed an integrated detection platform combining loop-mediated isothermal amplification (LAMP) with the CRISPR/Cas12b system in a single, one-step, one-pot reaction. The assay was optimized to function at a constant temperature of 58 °C and provides results within 45 minutes. The clinical performance of the system was evaluated using 22 clinical samples, and its diagnostic accuracy was compared with conventional PCR.

**Results:**

The LAMP-CRISPR/Cas12b assay demonstrated a limit of detection (LOD) of 14.77 copies per test, with no cross-reactivity observed against potential interfering nucleic acids, ensuring 100% specificity for HP. Clinical validation revealed a concordance rate of 90.91% (20/22) between the LAMP-CRISPR/Cas12b platform and conventional PCR, supporting the diagnostic reliability of the system.

**Discussion:**

The integrated LAMP-CRISPR/Cas12b platform represents a promising alternative for the rapid and sensitive detection of HP. It combines the simplicity and rapidity of LAMP with the specificity of CRISPR/Cas12b, offering a robust, cost-effective, and high\-sensitivity diagnostic tool without the need for complex instrumentation. The method shows great potential for use in point-of-care testing (POCT) and could significantly enhance clinical practice by facilitating timely diagnosis and treatment of HP-related diseases.

## Introduction

*Helicobacter pylori* (HP) is a globally prevalent gram-negative bacterium that colonizes the gastric mucosa (Malfertheiner et al., 2023). As an infectious agent, HP infections are predominantly asymptomatic in most cases (Wongphutorn et al., 2018). However, HP infections is strongly associated with severe gastritis-related diseases, including chronic gastritis, peptic ulcers, gastric mucosa‐associated lymphoid tissue lymphoma, and even gastric cancer (Zahir et al., 2024). With over half of the world’s population affected and its prevalence rising annually, HP infection poses a significant public health burden, implying the urgent need for rapid, accurate, and accessible diagnostic methods (Malfertheiner et al., 2012; Pak et al., 2020).

Currently, several diagnostic methods are available for HP detection, and endoscopy is employed in combination with histology, rapid urease test (RUT), microbial culture, and PCR-based tests using gastric biopsy specimens (Pohl et al., 2019). Alternative diagnostic approaches include the ^13^C-urea breath test, fecal antigen detection, and serological assays (Smith et al., 2014; Kuang et al., 2025). However, they are limited by prolonged turnaround times, labor-intensive procedures, suboptimal specificity, inability to distinguish active infections, invasiveness, reliance on specialized equipment, stringent sample transport conditions and handling requirements (Dincă et al., 2022; Fan et al., 2024; Moalla et al., 2024). Despite the availability of multiple diagnostic approaches, no single method has been unequivocally established as the gold standard for detecting HP, especially in epidemiological studies. Thus, ongoing efforts are focused on developing more reliable and universally applicable diagnostics for HP identification (Sulo and Sipkova, 2021).

Recent advances in CRISPR (clustered regularly interspaced short palindromic repeats)/Cas (CRISPR-associated proteins) systems have enabled highly specific nucleic acid detection (Gootenberg et al., 2017; Chen et al., 2018; Li et al., 2018; Kellner et al., 2019; Li et al., 2019). For HP diagnosis, an RPA (recombinase polymerase amplification)-CRISPR/Cas system has been developed (Qiu et al., 2021). Further refinements by Dai et al. introduced a one-tube RPA-CRISPR/Cas12a system, where RPA assay and the CRISPR/Cas detection occur sequentially via centrifugation, minimizing aerosol contamination risks (Dai et al., 2022). Despite these improvements, the method remains a two-step process requiring meticulous handing, which complicates its clinical adoption.

To overcome these limitations, we established a streamlined, a one-pot, one-step HP detection platform by integrating loop-mediated isothermal amplification (LAMP) with the CRISPR/Cas12b system, enabling rapid and contamination-free detection at a constant temperature (Huang et al., 2023). Targeting the cytotoxin-associated gene A (*CagA*), a key HP virulence factor (Peng et al., 2024), we optimized primer and sgRNA selection, as well as Cas12b and sgRNA concentrations, to maximize sensitivity and specificity. The performance of this one-step HP detection system was validated against conventional PCR using 22 clinical samples. Collectively, this study aims to develop a robust, rapid, and user-friendly diagnostic tool for HP detection, to offer significant advantages in clinical and point-of-care settings.

## Materials and methods

### Clinical samples

A total of 22 archived gastric mucosa samples were retrospectively collected from the Department of Pathology, Gastrointestinal Surgery at Renji Hospital, Shanghai Jiao Tong University School of Medicine. These residual diagnostic samples were obtained during routine clinical procedures. The study protocol was approved by the Ethics Committee of Renji Hospital, Shanghai Jiaotong University School of Medicine (LY2023-111-B), with waived informed consent due to the use of anonymized archival materials. Genomic DNA was extracted from the specimens using a commercial kit (TIANGEN, China) following the manufacturer’s instructions and stored at -20°C until analysis.

### LAMP primer and sgRNA design and selection

A highly conserved region of the *CagA* gene was selected as the detection target following sequence alignment analysis. The target sequence was cloned into a pUC57 plasmid to generate a recombinant standard. Ten LAMP primer sets (HP-LAMP-Primer-1 to HP-LAMP-Primer-10) were designed using the NEB LAMP primer design tool (https://lamp.neb.com ), with sequences detailed in **Supplementary Table 1**. Sixteen sgRNAs (sgRNA1 to sgRNA 16) targeting distinct regions of the *CagA* sequences were designed (**Table 1**). All primers were synthesized by Sangon Biotech (Shanghai, China), while sgRNAs were prepared using the Cas12b High Yield sgRNA Synthesis and Purification Kit (31904, Tolo Biotech, China) following the manufacturer’s protocol. 10× HP-LAMP primer mix was prepared by combining primers F3 and B3 (each at 2 uM final concentration), FIP and BIP (each at 16 uM final concentration), and LF and LB (each at 4 uM final concentration).

**Table 1:** The sequences of sgRNAs for the LAMP-CRISPR/Cas12b system.

sgRNAs	Sequence(5’-3’)
sgRNA1	GUCUAGAGGACAGAAUUUUUCAACGGGUGUGCCAAUGGCCACUUUCCAGGUGGCAAAGCCCGUUGAGCUUCUCAAAUCUGAGAAGUGGCACGGGAUAGGGGGUUGUAUGGU
sgRNA2	GUCUAGAGGACAGAAUUUUUCAACGGGUGUGCCAAUGGCCACUUUCCAGGUGGCAAAGCCCGUUGAGCUUCUCAAAUCUGAGAAGUGGCACUCAGGGAUAGGGGGUUGUAU
sgRNA3	GUCUAGAGGACAGAAUUUUUCAACGGGUGUGCCAAUGGCCACUUUCCAGGUGGCAAAGCCCGUUGAGCUUCUCAAAUCUGAGAAGUGGCACUCAUCAGGGAUAGGGGGUUG
sgRNA4	GUCUAGAGGACAGAAUUUUUCAACGGGUGUGCCAAUGGCCACUUUCCAGGUGGCAAAGCCCGUUGAGCUUCUCAAAUCUGAGAAGUGGCACUUUGUCAUCAGGGAUAGGGG
sgRNA5	GUCUAGAGGACAGAAUUUUUCAACGGGUGUGCCAAUGGCCACUUUCCAGGUGGCAAAGCCCGUUGAGCUUCUCAAAUCUGAGAAGUGGCACUUUCUUUGUCAUCAGGGAUA
sgRNA6	GUCUAGAGGACAGAAUUUUUCAACGGGUGUGCCAAUGGCCACUUUCCAGGUGGCAAAGCCCGUUGAGCUUCUCAAAUCUGAGAAGUGGCACGCUUUUUCUUUGUCAUCAGG
sgRNA7	GUCUAGAGGACAGAAUUUUUCAACGGGUGUGCCAAUGGCCACUUUCCAGGUGGCAAAGCCCGUUGAGCUUCUCAAAUCUGAGAAGUGGCACAAAAACUCUGCUUUUUCUUU
sgRNA8	GUCUAGAGGACAGAAUUUUUCAACGGGUGUGCCAAUGGCCACUUUCCAGGUGGCAAAGCCCGUUGAGCUUCUCAAAUCUGAGAAGUGGCACGCAGAUUUCAAAAACUCUGC
sgRNA9	GUCUAGAGGACAGAAUUUUUCAACGGGUGUGCCAAUGGCCACUUUCCAGGUGGCAAAGCCCGUUGAGCUUCUCAAAUCUGAGAAGUGGCACUUUGGCAGAUUUCAAAAACU
sgRNA10	GUCUAGAGGACAGAAUUUUUCAACGGGUGUGCCAAUGGCCACUUUCCAGGUGGCAAAGCCCGUUGAGCUUCUCAAAUCUGAGAAGUGGCACGCCAAACAAUCUUUUGCAGG
sgRNA11	GUCUAGAGGACAGAAUUUUUCAACGGGUGUGCCAAUGGCCACUUUCCAGGUGGCAAAGCCCGUUGAGCUUCUCAAAUCUGAGAAGUGGCACCAGGAAUCAUUAUAGGGAAU
sgRNA12	GUCUAGAGGACAGAAUUUUUCAACGGGUGUGCCAAUGGCCACUUUCCAGGUGGCAAAGCCCGUUGAGCUUCUCAAAUCUGAGAAGUGGCACUUAUAGGGAAUCAAAUCCGA
sgRNA13	GUCUAGAGGACAGAAUUUUUCAACGGGUGUGCCAAUGGCCACUUUCCAGGUGGCAAAGCCCGUUGAGCUUCUCAAAUCUGAGAAGUGGCACUAGGGAAUCAAAUCCGAACG
sgRNA14	GUCUAGAGGACAGAAUUUUUCAACGGGUGUGCCAAUGGCCACUUUCCAGGUGGCAAAGCCCGUUGAGCUUCUCAAAUCUGAGAAGUGGCACAAUCCGAACGGAUCAAAAGU
sgRNA15	GUCUAGAGGACAGAAUUUUUCAACGGGUGUGCCAAUGGCCACUUUCCAGGUGGCAAAGCCCGUUGAGCUUCUCAAAUCUGAGAAGUGGCACGAACGGAUCAAAAGUUCAUG
sgRNA16	GUCUAGAGGACAGAAUUUUUCAACGGGUGUGCCAAUGGCCACUUUCCAGGUGGCAAAGCCCGUUGAGCUUCUCAAAUCUGAGAAGUGGCACAAAGAUUCAUCAAACACGCC

### Optimization of one-step LAMP-CRISPR/Cas12b assay

The 25 μL reaction system contained:

2.5 μL 10 × LAMP buffer (25102, Tolo Biotech, China),

1.0 μL dNTP mix (25 mM, DN32, Hongene, China),

1.5 μL MgSO_4_ (100 mM, B1003S, NEB, USA),

2.5 μL 10× HP-LAMP primer mix,

6 μL Glycine (2 M, A610235, Sangon, China),

1.25 μL HOLMES ssDNA-Reporter-FAM (10 uM, 31101, Tolo Biotech, China),

0.625 μL AapCas12b (10 uM, 32118, Tolo Biotech, China),

1 μL Bst DNA polymerase (25102, Tolo Biotech, China),

0.625 μL HP-sgRNA (10 uM),

5.5 μL Nuclease-free water (R1600, Solarbio, China),

2.5 μL template DNA (200 copies/μL HP recombinant plasmid).

Optimization parameters included:

1. Cas12b and sgRNA (1:1) final concentrations (250 nM, 500 nM, 750 nM, and 1000 nM).
2. Reaction temperature gradient (55-60°C in 1°C increments).
3. Four fluorescent HOLMES ssDNA reporters: PA8-Repoter (5’-/6-FAM/AAAAAAAA/BHQ1/-3’), PT8-Repoter (5’-/6-FAM/TTTTTTTT/BHQ1/-3’), PC8-Repoter (5’-/6-FAM/CCCCCCCC/BHQ1/-3’), and PG8-Repoter (5’-/6-FAM/GGGGGGGG/BHQ1/-3’).

### Analytical sensitivity and specificity

The limit of detection (LOD) was determined using serial dilutions of HP-*CagA* recombinant plasmid (100, 50, 25, and 12.5 copies/test). Copy numbers were calculated based on plasmid molecular weight (1 ng ≈ 7.6×10^9^ copies). Each concentration was tested in octuplicate, with LOD determined via sigmoidal curve analysis.

Specificity was evaluated against eight non-HP strains: *Proteus mirabilis, Pseudomonas aeruginosa, Salmonella sonnei, Candida albicans, Enterococcus faecalis, Escherichia coli, Acinetobacter*, and *Diplococcus pneumoniae*. Extracted nucleic acids were tested alongside controls:

Positive control (PC): HP recombinant plasmid,

Negative control (NC): Nuclease-free water.

All reactions were performed in triplicate.

### Clinical validation

The assay’s diagnostic performance was validated using 22 human clinical gastric mucosa samples, with conventional quantitative polymerase chain reaction (qPCR) as the reference method. All these 22 samples were confirmed as HP-positive by RUT in Renji Hospital. Parallel testing was conducted using both the LAMP-CRISPR/Cas12b system and qPCR, with results compared to determine concordance rates.

#### qPCR assay

The qPCR primers and probe were designed as follows:

Forward primer: CCGTCTAAAATCAACACCCGA;

Reverse primer: TCAAACACGCCCATGAACTT;

Probe: 5’FAM-AGGGAATCAAATCCGAACGG-3’BHQ1.

All oligonucleotides were designed and synthesized by Sangon Biotech (Shanghai, China). The reaction was performed using High Affinity Hot StartTaq (TIANGEN, Beijing, China) on a Quant Studio 5 Real-Time PCR system (Applied Biosystems, USA). The thermal cycling conditions were: 95°C for 5min of initial denaturation, 45 cycles of amplification (95°C 15s), and 54°C for 30s (for FAM signal collection).

### Statistical analysis

All statistical analyses were performed using Graphpad Prism software (version 8.2). Each experiment was conducted in triplicate, and the data were expressed as mean ± standard deviation (SD). The LOD of present method was predicated by using the Sigmoid function according to the positive results of each dose, at the probability of 95%. Statistical significance was determined using Student’s t-test, with significance thresholds set at *P < 0.05, **P < 0.01, ***P < 0.001, ****P < 0.0001.

## Results

### Workflow of the one-step LAMP-CRISPR/Cas12b system

**Figure 1:**
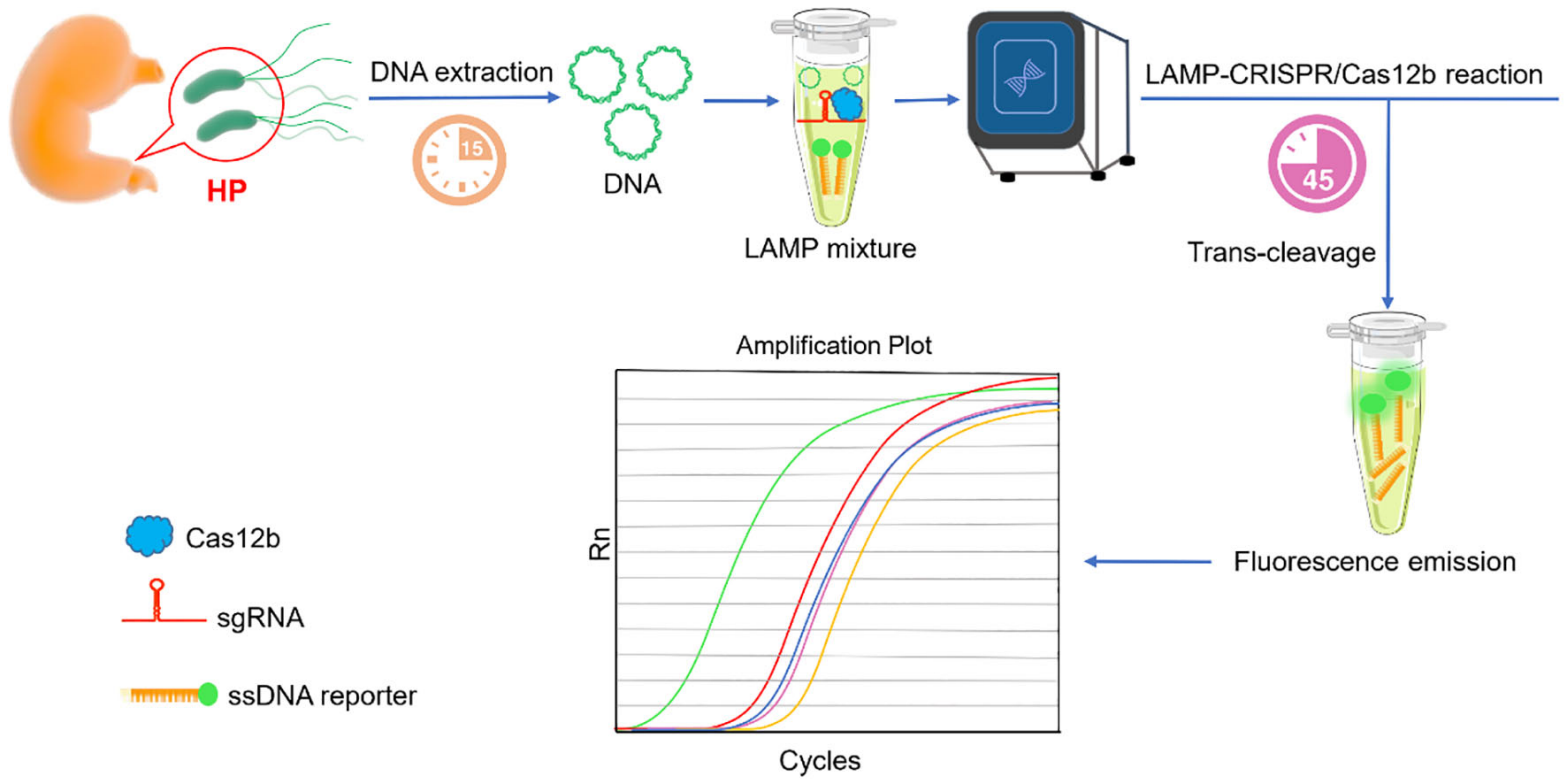
Schema illustration of the one-step LAMP-CRISPR/Cas12b platform for HP detection. After nucleic acid samples extraction, the template was directly added to reaction. Both the LAMP amplification and CRISPR/Cas12b detection were simultaneously undergoing. A remarkable fluorescence can be detected in the presence of the target sequence.

In this study, the entire detection process was performed in a fully integrated one-pot, one-step reaction, and the detailed schematic is illustrated in **Figure 1**. Following nucleic acid extraction and LAMP-mediated amplification of HP genomic targets, CRISPR/Cas12b-mediated detection was performed using an HP-specific sgRNA. The reaction was carried out at a constant temperature of 58°C. In target-positive samples, Cas12b’s cis-cleavage activity first processes the target sequence, which leads to the activation of trans-cleavage activity of Cas12b. By virtue of the trans-cleavage activity, Cas12b degrades the FAM-labeled single-stranded DNA (ssDNA) reporter probe, followed by a remarkable fluorescence signal release. Eventually, the fluorescence signal was monitored in real-time on a Fluorescent Quantitative PCR instrument. The complete workflow required only 45 minutes from sample to result, eliminating the need for intermediate handing steps.

### LAMP primer and sgRNA selection

**Figure 2:**
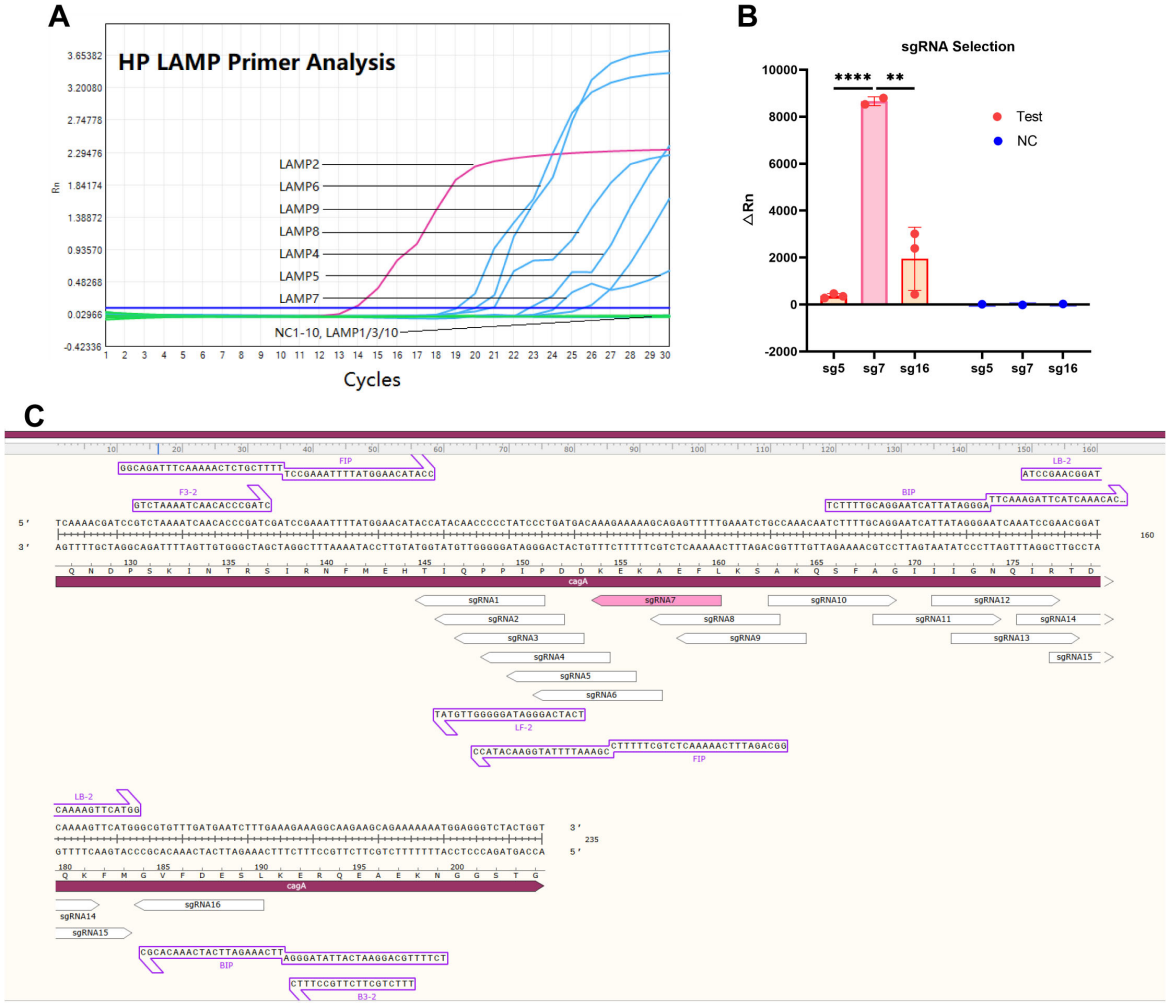
The LAMP primer and Cas12b sgRNA selection for one-step LAMP-CRISPR/Cas12b platform. **(A)** The amplification curve of the LAMP assay by using different LAMP primers. **(B)** The fluorescence signal value of performing the LAMP-CRISPR/Cas12b system using sgRNA 5, sgRNA7, and sgRNA16. **(C)** The nucleotide sequence of the expected LAMP product, and the position of the sgRNA target site. * indicates P < 0.05, and **** indicates P < 0.0001.

Using the well-characterized virulence factor *CagA* (Gene ID: 93236896) as the detection target of HP (Mahant et al., 2024; Singh et al., 2024), we evaluated 10 LAMP primers sets (HP-LAMP-Primer-1 to HP-LAMP-Primer-10) targeting conserved *CagA* regions. As illustrated in **Figure 2A**, six primer sets showed successful amplification, with HP-LAMP-Primer-2 demonstrating optimal efficiency.

According to the yielded △Rn values, HP-sgRNA5, -sgRNA7 and -sgRNA16 were considered as the three high-performing sgRNAs, and HP-sgRNA7 exhibited superior detection capability (**Figure 2B**) (**Supplementary Table 2**-sgRNA). Consequently, HP-LAMP-Primer-2 was selected for subsequent target amplification and HP-sgRNA7 was chosen for target sequence detection. **Figure 2C** demonstrated the expected LAMP product, including its nucleotide sequence and the position of the sgRNA target site. The protospacer adjacent motif (PAM) sequences were defined as 5’-TTN-3’ (N = A, T, C, or G) and the 5’-CTN-3’ (N = A, C, or G) located at the 5’ end of the protospacer.

### Optimization of the reaction conditions

**Figure 3:**
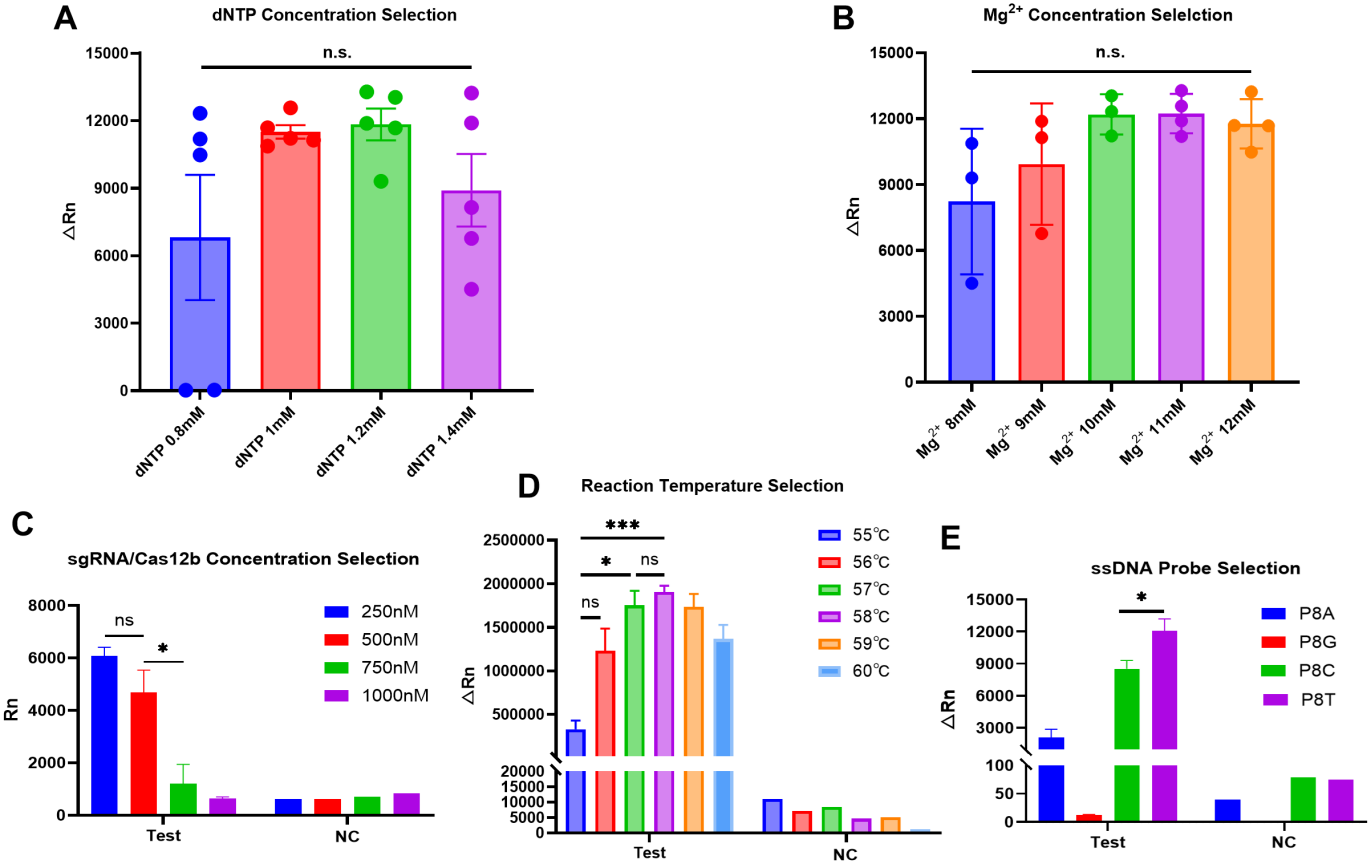
Optimization of the one-step HP LAMP-CRISPR/Cas12b reaction. **(A, B)**. The selection for dNTP concentration (0.8,1,1.2 and 1.4mM) and Mg^2+^ concentration (8, 9,10,11 and 12 mM); n.s., no significance. **(C)**. The paired dose selection of sgRNA/Cas12b ranged from 250 nM,500 nM,750 nM, and 1000 nM respectively. **(D)**. The reaction temperature was selected from 55°C to 60°C by 1°C increments. **(E)**. The selection of ssDNA reporters, including PA8, PG8, PC8 and PT8. * indicates P < 0.05, and *** indicates P < 0.001. PC8, Polycytosine-8-nt Fluorescent Reporter; PA8, Polyadenine-8-nt Fluorescent Reporter; PG8, Polyguanine-8-nt Fluorescent Reporter; PT8, Polythymine-8-nt Fluorescent Reporter.

To optimize the performance of the one-step LAMP-CRISPR/Cas12b reaction, several parameters were systematically evaluated, including the dNTP concentration, MgSO_4_ (Mg^2+^) concentration, sgRNA/Cas12b (1:1) concentration, reaction temperature, and reporter probe. As demonstrated in **Figure 3A**, fluorescence signal intensity reached its peak at a dNTP concentration of 1mM. Parallel optimization revealed that 11 mM MgSO_4_ provided optimal reaction kinetics (**Figure 3B**). Furthermore, the highest fluorescence signal was obtained when using 250 nM sgRNA/Cas12b complex (**Figure 3C**).

A comprehensive temperature optimization was performed across a gradient from 55-60°C in 1°C increments. Systematic analysis of reaction kinetics revealed 5 8°C as the optimal temperature, indicating both robust amplification efficiency and minimal non-specific signal generation (**Figure 3D**). Subsequently, we evaluated four distinct ssDNA reporter probes (PA8-, PC8-, PG8-, and PT8-Reporter) to identify the most effective detection system. Comparative fluorescence analysis demonstrated that PT8-Reporter yielded significantly higher signal intensity with superior signal-to-noise characteristics, establishing it as the optimal choice for subsequent experiments (**Figure 3E**).

### Sensitivity and specificity analysis of the LAMP-CRISPR/Cas12b system

**Figure 4:**
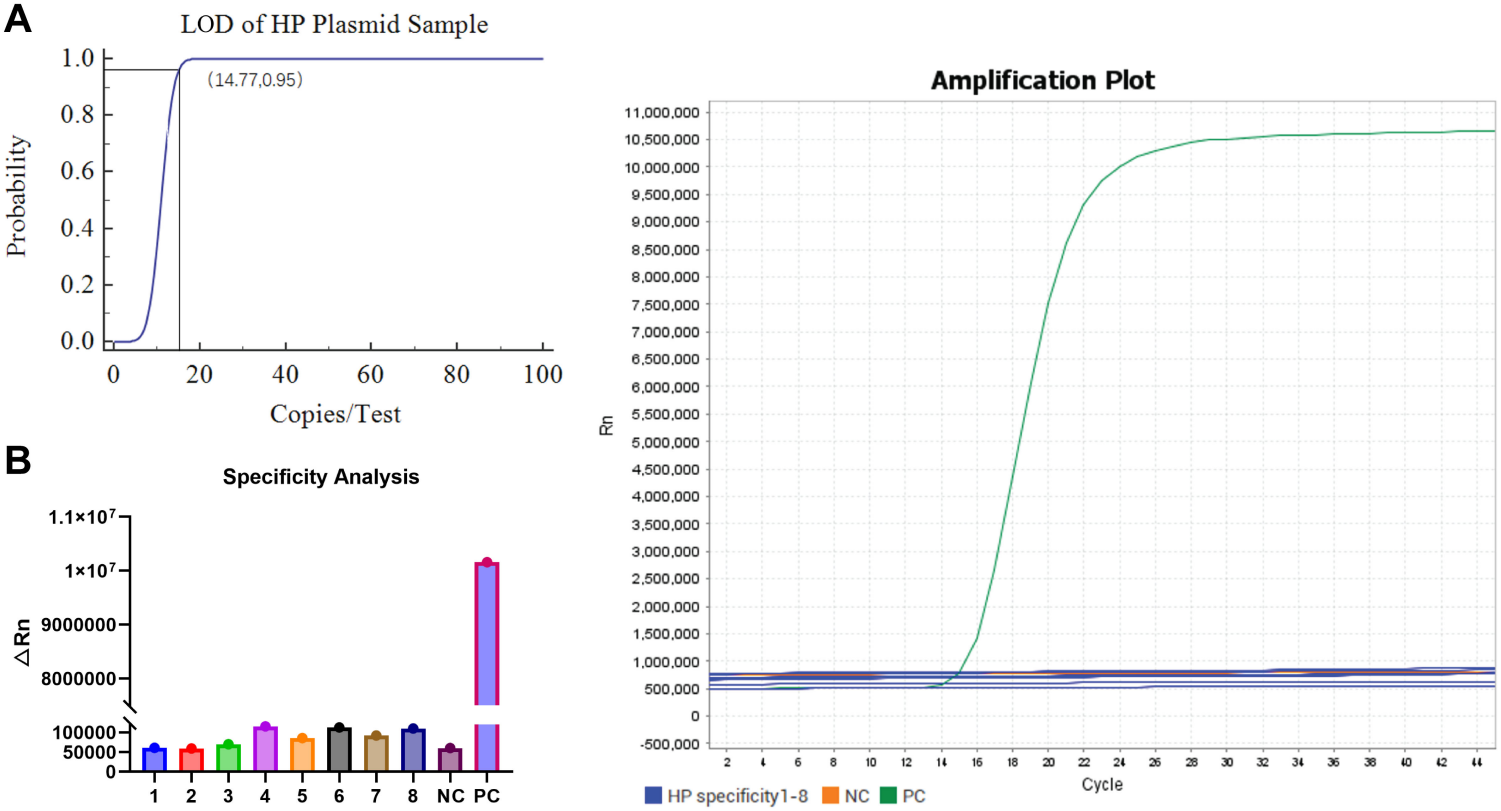
Sensitivity and specificity analysis of one-step LAMP-CRISPR/Cas12b HP detection system. **(A)** The LOD analysis of the LAMP-CRISPR/Cas12b HP detection system by diluting the recombinant plasmid. **(B)** The specificity analysis of the LAMP-CRISPR/Cas12b HP detection system using eight interferes samples, including *Proteus mirabilis, Pseudomonas aeruginosa, Salmonella sonnei, Candida albicans, Enterococcus faecalis, Escherichia coli, Acinetobacter*, and *Diplococcus pneumoniae*. The recombinant plasmid of HP CagA was served as the positive control (PC) and nuclease-free water was used as negative control (NC).

The LOD was systematically evaluated using serial dilutions of recombinant plasmids containing the HP *CagA* target sequence. Quantitative analysis revealed consistent detection across eight replicates at concentrations of 100, 50, and 25 copies/test, with 75% detection efficiency (6/8 replicates) at the 12.5 copies/test level. Sigmoidal curve fitting established an LOD of 14.77 copies/test at 95% detection probability (**Figure 4A**), confirming the assay’s high sensitivity.

Specificity assessment included eight clinically relevant bacterial and fungal species: *Proteus mirabilis, Pseudomonas aeruginosa, Salmonella sonnei, Candida albicans, Enterococcus faecalis, Escherichia coli, Acinetobacter*, and *Diplococcus pneumoniae*. The system exhibited exceptional discrimination, with significant fluorescence signals exclusively observed for HP-positive controls (PC). No cross-reactivity was detected with any non-target organisms or negative controls (NC) (**Figure 4B**), suggesting 100% analytical specificity for HP detection.

### Validation of the LAMP-CRISPR/Cas12b for HP detection using human clinical samples

The results of performance validation showed that the LAMP-CRISPR/Cas12b system detected 18 HP-positive and 4 HP-negative samples in 22 human clinical gastric mucosa samples (all of them were confirmed as HP-positive by RUT), while PCR identified 20 HP-positive and 2 HP-negative samples. The two discordant cases (PCR-positive/CRISPR-negative) likely represent samples with bacterial loads near the assay’s detection limit of the LAMP-CRISPR/Cas12b assay, as evidenced by their significantly higher PCR cycle threshold (Ct) values (32 and 34.8) compared to the average Ct value of 24.7 observed in other positive samples (**Supplementary Table 2**-clinical validation). Importantly, no false positives were observed, demonstrating the method’s high specificity in clinical applications. The overall concordance rate between the two methods reached 90.91% (20/22, **Table 2**). The results proved that the LAMP-CRISPR/Cas12b platform offers high reliability comparable to standard PCR and excellent specificity for HP detection in complex clinical samples.

**Table 2:** Comparison between performance of the CRISPR and Sequence for HP detection for clinical samples.

CRISPR	qPCR		Sum	Sensitivity	Specificity	Concordance
	No. positive	No. negative				
Positive	18	0	18	90.00%	100.00%	90.91%
Negative	2	2	4			
Total	20	2	22			

## Discussion

HP is a leading etiological agent of gastritis, gastric cancer, and other related gastrointestinal disorders, presents a significant diagnostic challenge as most infected individuals remain asymptomatic (Godbole et al., 2020; Kuraoka et al., 2024). This clinical reality underscores the urgent need for novel detection methods that combine rapidity, accuracy, and user-friendliness. Addressing this need, we reported the development of a one-step, one-pot HP detection system that integrates LAMP with the CRISPR/Cas12b platform. Our method demonstrates exceptional reliability, clinical practicality, and excellent specificity when applied to gastric mucosa specimens, achieving complete detection within a remarkable 45-minute timeframe through a streamlined workflow. Furthermore, compatibility with lyophilized reagent preparation significantly enhances its stability and operational convenience for clinical use. These performance characteristics establish its strong potential as a robust diagnostic solution, particularly in settings requiring rapid results with minimal technical complexity.

The exceptional sensitivity of our system stems from the synergistic combination of isothermal amplification (Ivanov et al., 2022) and CRISPR/Cas technology, which has previously shown promise in enhancing HP detection via lateral flow assays (Dong et al., 2025). The Cas12b component provides an additional layer of specificity through its dual functionality: collateral cleavage activity upon target recognition (Li et al., 2019; Hao et al., 2024) and remarkable single-base mismatch discrimination capability (Varshney et al., 2021). Our validation studies demonstrated a LOD of 14.77 copies/test in HP-*CagA* recombinant plasmids, with excellent discriminatory power against other microorganisms. Above all, LAMP-CRISPR/Cas12b platform achieved diagnostic reliability comparable to standard PCR while offering superior specificity for nucleic acid-based HP identification. Nevertheless, our LAMP-CRISPR/Cas12b system failed to detect the HP-positive samples with low bacterial loads, suggesting that further optimization of amplification conditions is necessary for such cases. Additionally, this study is limited by its relatively small cohort of clinical samples (n=22). To establish a more conclusive assessment of the assay’s clinical diagnostic performance, future validation with larger sample sizes is warranted. Such studies would better elucidate its clinical utility and unlock its full potential for real-world diagnostic applications.

When compared to existing technologies, our system shows distinct advantages. While previous studies reported an RPA-CRISPR/Cas12a platform with 2 ng/µL sensitivity in human saliva samples (Zhu et al., 2023) and 5 copies/µL LOD in stool specimens (Qiu et al., 2021), our LAMP-CRISPR/Cas approach demonstrates comparable performance for HP detection in clinical gastric mucosa samples while offering potential technical benefits. The multiple primers in LAMP may enhance specificity, and the broader adoption of LAMP-based versions of CRISPR/Cas diagnostics in the field suggests greater practical utility (Rolando et al., 2024). Although currently validated for gastric mucosa samples, we are actively working to adapt this platform for non-invasive sampling. Thus, these developments will further facilitate the translation of this technology toward automatic point-of-care testing (POCT) and *in vitro* diagnostic devices.

Our selection of the *CagA* gene as the detection target represents another strategic advantage, as this virulence factor is present in nearly all HP strains and serves as an established biomarker for gastric cancer risk in East-Asian countries (Takahashi-Kanemitsu et al., 2020; Duan et al., 2025). While previous work has employed *CagA* as the target gene for HP detection and typing using RPA-CRISPR/Cas12a technology (Dai et al., 2022; Zhu et al., 2023), our study represents the first successful implementation of a LAMP-CRISPR/Cas12b platform targeting this gene, thereby enabling simultaneous HP identification and gastric cancer risk assessment.

In light of the current study’s limitations, we outline the following strategies to guide further investigations: (1) Comprehensive evaluation across larger cohorts, diverse sample types and clinical settings is required to fully characterize the system’s reliability and stability; (2) The current reliance on gastric mucosa samples highlights the need for ongoing development of non-invasive alternatives to improve patient compliance and clinical utility; and (3) This study employed a specialized fluorescent quantitative PCR instrument for system establishment due to its high precision in temperature control and signal detection. Future research will focus on developing simplified tools for temperature adjustment and signal collection, along with visual detection devices and test strips.

In conclusion, we have developed an original one-step LAMP-CRISPR/Cas12b system that demonstrates exceptional sensitivity, specificity, and POCT compatibility for HP detection. Beyond its immediate application for HP diagnosis, this platform establishes a versatile framework that could be adapted for identification of other pathogens, representing a significant advancement in molecular diagnostic technologies.Data availability statement

The raw data supporting the conclusions of this article will be made available by the authors, without undue reservation.
