# Supplementary material for: A rapid and accurate method for Helicobacter pylori detection via integrating LAMP assay with CRISPR/Cas12b detection by one-step in one-pot
Source: Front Cell Infect Microbiol. 2025 Aug 26;15:1611134. doi: 10.3389/fcimb.2025.1611134 (PMC12417528; doi:10.3389/fcimb.2025.1611134)
Supplement: Supplementary file 1 [file Table1.docx]

Supplementary Table 1. The LAMP primer sequence for HP *CagA* gene

| Primer | | Sequence（5’-3’） |
| --- | --- | --- |
| HP-LAMP-Primer-1 | HP_cagA-F3-1 | AACGATCCGTCTAAAATCAAC |
|  | HP_cagA-B3-1 | TTTCTGCTTCTTGCCTTTC |
|  | HP_cagA-FIP-1 | GCTTTTTCTTTGTCATCAGGGATAGACCCGATCGATCCGAAAT |
|  | HP_cagA-BIP-1 | TGAAATCTGCCAAACAATCTTTTGCAAACACGCCCATGAACTT |
|  | HP_cagA-LB-1 | TAGGGAATCAAATCCGAACGGATCA |

| Primer | | Sequence（5’-3’） |
| --- | --- | --- |
| HP-LAMP-Primer-2 | HP_cagA-F3-2 | GTCTAAAATCAACACCCGATC |
|  | HP_cagA-B3-2 | TTTCTGCTTCTTGCCTTTC |
|  | HP_cagA-FIP-2 | GGCAGATTTCAAAAACTCTGCTTTTTCCGAAATTTTATGGAACATACC |
|  | HP_cagA-BIP-2 | TCTTTTGCAGGAATCATTATAGGGATTCAAAGATTCATCAAACACGC |
|  | HP_cagA-LF-2 | TCATCAGGGATAGGGGGTTGTAT |
|  | HP_cagA-LB-2 | ATCCGAACGGATCAAAAGTTCATGG |

| Primer | | Sequence（5’-3’） |
| --- | --- | --- |
| HP-LAMP-Primer-3 | HP_cagA-F3-3 | ACCCGATCGATCCGAAAT |
|  | HP_cagA-B3-3 | AATCCCCACCAGTAGACC |
|  | HP_cagA-FIP-3 | TGGCAGATTTCAAAAACTCTGCTAACATACCATACAACCCCCT |
|  | HP_cagA-BIP-3 | CAAATCCGAACGGATCAAAAGTTCCCATTTTTTTCTGCTTCTTGC |
|  | HP_cagA-LB-3 | TGGGCGTGTTTGATGAATCTTTG |

| Primer | | Sequence（5’-3’） |
| --- | --- | --- |
| HP-LAMP-Primer-4 | HP_cagA-F3-4 | CAAAGATTCATCAAACACGC |
|  | HP_cagA-B3-4 | GGTGTCCCATCAAAACGA |
|  | HP_cagA-FIP-4 | GCCAAACAATCTTTTGCAGGAATCCCATGAACTTTTGATCCGTT |
|  | HP_cagA-BIP-4 | AACTCTGCTTTTTCTTTGTCATCAGACCCGATCGATCCGAAAT |
|  | HP_cagA-LB-4 | GATAGGGGGTTGTATGGTATGTTCC |

| Primer | | Sequence（5’-3’） |
| --- | --- | --- |
| HP-LAMP-Primer-5 | HP_cagA-F3-5 | CTCCATTTTTTTCTGCTTCTTG |
|  | HP_cagA-B3-5 | GTCTAAAATCAACACCCGATC |
|  | HP_cagA-FIP-5 | TGCAGGAATCATTATAGGGAATCAATCTTTCAAAGATTCATCAAACAC |
|  | HP_cagA-BIP-5 | GTTTGGCAGATTTCAAAAACTCTGCTTTTATGGAACATACCATACAACC |
|  | HP_cagA-LF-5 | GAACGGATCAAAAGTTCATGGGC |

| Primer | | Sequence（5’-3’） |
| --- | --- | --- |
| HP-LAMP-Primer-6 | HP_cagA-F3-6 | ATGGATCCTAATTACAAGTTCAA |
|  | HP_cagA-B3-6 | ATTGTAGCCACATTGTTACC |
|  | HP_cagA-FIP-6 | CAGGTTCTATGCCATCATGACTCTATTGATTCACAATAACACTCTGTC |
|  | HP_cagA-BIP-6 | AAGTTTCATTATTGTATGCGGGCAGGTCTTTATAGCCAACGGTG |
|  | HP_cagA-LB-6 | TGGTTTTGGAGACAAGCACGA |

| Primer | | Sequence（5’-3’） |
| --- | --- | --- |
| HP-LAMP-Primer-7 | HP_cagA-F3-7 | GCATAGAACCTGAAAAAGTTTC |
|  | HP_cagA-B3-7 | TGTGAGTTGGTCTTCTTTGT |
|  | HP_cagA-FIP-7 | TGTTGGTCTTTATAGCCAACGGGGCAATGGTGGTTTTGGA |
|  | HP_cagA-BIP-7 | TAATGTGCATATGAAAAACGGCAGGGGTTGTTAATCCCTTTCTCA |
|  | HP_cagA-LF-7 | GGCGTTCCAATCGTGCTTGT |

| Primer | | Sequence（5’-3’） |
| --- | --- | --- |
| HP-LAMP-Primer-8 | HP_cagA-F3-8 | AAAAGTTTCATTATTGTATGCGG |
|  | HP_cagA-B3-8 | GAGCCTGTGAGTTGGTCT |
|  | HP_cagA-FIP-8 | CCACATTGTTACCTTGTTGGTCTTTGTTTTGGAGACAAGCACG |
|  | HP_cagA-BIP-8 | TGCATATGAAAAACGGCAGTGGTCTTTGTAGAGATAAAAACTAGGGT |
|  | HP_cagA-LF-8 | AGCCAACGGTGGCGTTC |
|  | HP_cagA-LB-8 | ATAGCAGGTGGTGAGAAAGGGAT |

| Primer | | Sequence（5’-3’） |
| --- | --- | --- |
| HP-LAMP-Primer-9 | HP_cagA-F3-9 | GCCGTTTTTCATATGCACATT |
|  | HP_cagA-B3-9 | TGATTCACAATAACACTCTGTC |
|  | HP_cagA-FIP-9 | GACAAGCACGATTGGAACGCTATTGTAGCCACATTGTTACC |
|  | HP_cagA-BIP-9 | CACCATTGCCCGCATACAATTTCTGTGTTAATAGGGAGTCATG |
|  | HP_cagA-LF-9 | ACCGTTGGCTATAAAGACCAACAA |

| Primer | | Sequence（5’-3’） |
| --- | --- | --- |
| HP-LAMP-Primer-10 | HP_cagA-F3-10 | GCTATGACTAAGCCACTGC |
|  | HP_cagA-B3-10 | ACACTCTGTCTTCTGTGTT |
|  | HP_cagA-FIP-10 | ACGCCACCGTTGGCTATAAAGCACATTAATTATTGTAGCCACAT |
|  | HP_cagA-BIP-10 | TCCAATCGTGCTTGTCTCCAAAGGGAGTCATGATGGCATAG |
|  | HP_cagA-LB-10 | ACCACCATTGCCCGCATAC |
